# Determining the Authenticity of Shark Meat Products by DNA Sequencing

**DOI:** 10.3390/foods9091194

**Published:** 2020-08-28

**Authors:** Patrizia Marchetti, Anna Mottola, Roberta Piredda, Giuseppina Ciccarese, Angela Di Pinto

**Affiliations:** 1 Department of Veterinary Medicine, University of Bari Aldo Moro, Prov. le Casamassima, km 3, 70010 Valenzano (Bari), Italy; patrizia.marchetti@uniba.it (P.M.); anna.mottola@uniba.it (A.M.); 2 Stazione Zoologica Anton Dohrn, Villa Comunale, 80121 Napoli, Italy; robpiredda@gmail.com; 3 Istituto Zooprofilattico Sperimentale della Puglia e della Basilicata, S.S. 7 ter, km 73, Zona Industriale, 73012 Campi Salentina (LE), Italy; giuseppina.ciccarese@izspb.it

**Keywords:** shark meat products, DNA sequencing, species identification, food authenticity, food safety, mislabeling, fishery regulations, environmental sustainability, traceability

## Abstract

Given that the global shark meat market is poised to grow in future years, the aim of this study was to use DNA sequencing of the cytochrome c oxidase I (COI) and NADH dehydrogenase subunit 2 (NADH2) mitochondrial genes to examine the market of shark meat products in Italy. This made it possible to analyze patterns of species utilization and commercialization of threatened, endangered and/or prohibited species, focusing on fraudulent activities in the shark food chain in order to propose seafood safety and environmental sustainability solutions. The study shows that the labeling of shark meat products generally lacks comprehensive information, thus making it difficult for consumers to make informed purchasing decisions and fails to comply with European Union (EU) legislation regarding seafood labelling. Molecular investigation reveals a high mislabeling rate (45.4%), highlighting widespread use of cheaper species either in order to replace species that are better known and more popular, or else in order to sell various threatened species. Considering that seafood mislabeling can circumvent the management of sustainable fisheries and facilitate Illegal, Unreported and Unregulated (IUU) fishing, the routine use of genetic analysis should be encouraged among control and enforcement agencies in order to implement effective management measures. This would help to build a species-specific reporting system for all catches, and enhance control measures, in order to prevent illegal activities connected with shark catches and trade around the world.

## 1. Introduction

Sharks are fish of great commercial importance, highly appreciated and widely consumed in many countries [1]. Although a large number of shark products, such as fins, cartilage, leather, liver oil, jaws and teeth, are already being widely traded [2,3,4], the global shark meat market looks set to grow in future years, with shark meat products commercialized in various forms, such as whole, fresh or frozen, tails, slices, fillets or minced, smoked, salted and dried meat [1,5].

Species substitutions regularly occur in this sector and are recognized as being a significant global issue. Unintentional mislabeling occurs when species are misidentified or when information is lost at some point along the supply chain. Indeed, variations of derivative products associated with morphological similarities between different species and the worldwide increase in commerce and processing of sharks have favored the potential for species substitution with and among shark species, which are often misgraded and mislabeled [5,6,7]. Sometimes, mislabelling [8] can be related to inconsistencies in the various commercial designations, drawn up and accepted in each EU country, including accepted local or regional names and thus to adventitious assignment to a species with a common vernacular name. Intentional mislabeling is often practiced in order to replace expensive, popular and high-value species with cheaper ones. Specifically, substitution species linked to the intentional replacement of species of greater economic value with species of a lesser value could be ascribed to the low commercial value of shark meat compared to other fish species [9,10]. Intentional mislabeling allows sellers to label cheaper species as more profitable ones, or to disguise the sale of species. Indeed, illegal over-quota catches and catches of protected species may also be mislabeled and placed on the market [11].

Currently, many shark populations have declined globally and are considered threatened or endangered by the International Union for the Conservation of Nature (IUCN) Red List of Threatened Species [12], due to a combination of anthropogenic stressors, such as habitat degradation, overfishing, illegal fishing and recreational fishing [13]. The consequence of this over-exploitation is the thriving illicit sale of endangered species at local markets, a trade which has a significant environmental impact [14,15]. However, the illegal trading of endangered and protected species is often due to unintentionally incorrect morphological identification, considering that the morphological identification of fish depends to a great extent on personal experience, but also on abiotic factors such as environmental perturbations, which can affect body shape, skin color and other external characteristics [16], leading inevitably to controversy and misidentification [17]. Given that illegal practices can negatively impact food safety and consumer confidence in the seafood supply, there is clearly a need for efficient schemes to guarantee and reinforce the traceability of fish and fish products from sea to plate, in order to update and reinforce fisheries control and strengthen schemes to fight intentional and unintentional species substitution. Consequently, there is an increasing need for cheap, rapid, reliable testing to determine the taxonomic identity of commercialized shark meat products. Identification of species based on a DNA barcoding approach is a consolidated strategy in biodiversity assessments, and its applications to food have also been growing in recent years. It is based on the use of a short and standardized DNA-barcode region containing sufficient sequence variation to distinguish unambiguously between species. Thanks to the quite easy procedures of amplification, sequencing, analyses and the amount of molecular variation displayed (lower intra-specific variation and higher inter-species variation able to discriminate even between sister species), the mitochondrial cytochrome c oxidase I (COI) gene was identified as the official barcode region for metazoan groups [18]. This led to an enormous increase in the use of this marker for several purposes with more reference sequences becoming available and the generation of a curated public database [19]. Indeed, molecular identification helps overcome difficulties related to the classical approach, such as the lack of morphological diagnostic characters both in adults and in other life-stages, the shortage of trained taxonomists, but also the possibility to identify species from fragments or parts of the body.

Fish represent half of all vertebrate species with about 35,000 species including marine and freshwater [20]. For fish, the research project Fish Barcode of Life Initiative (FISH-BOL), launched in 2005 [21], is probably the most important initiative that not only increased the amount of reference sequences on a global scale, but also provided laboratory protocols, alternative primer cocktails and suggestions to overcome failures [22,23]. Identification of fish species by DNA barcoding has been applied in several contexts [24,25,26,27] and has also been used to certificate the authenticity of fish species by detecting species substitution on commercial markets [9,28,29,30]. Indeed, in the fields of seafood safety, DNA barcoding has earned itself a central role, demonstrating its potential for fast and reliable identification of frauds and is a valid method used by the US Food and Drug Administration for species identification of fish [31].

However, due to species hybridization, introgression or incomplete lineage sorting, in some fishes including some sharks [22,32] as well in other taxa [33], the official DNA-barcode region COI does not allow for rigorous species discrimination. Moreover, the variability often present in conserved priming regions has forced researchers to develop and apply alternative primers and marker regions. The most common alternative or complementary regions, used for fishes, include the mitochondrial cytochrome b (Cytb) [34,35], mitochondrial 16S ribosomal RNA (16S rRNA) [7,36], mitochondrial 12S ribosomal RNA (12S rRNA) [37,38] and the mitochondrial NADH dehydrogenase subunit 2 (NADH2) [39,40,41,42,43]. The latter has been reported as a fast-evolving region [39,40,42] and has also been found to be decisive in the identification of *Mustelus higmani*, which does not have COI reference sequences deposited in any public repository [44].

Considering that several studies use multiple markers [45,46,47,48,49,50], thus highlighting the utility of using multiple markers for species identification [44,51], this study aims to use the combination of DNA sequencing of the cytochrome c oxidase subunit 1 gene (COI) and the NADH dehydrogenase subunit 2 gene (NADH2), to obtain an accurate genetic identification of prepared shark meat products in Italy. Firstly, similarity and phylogenetic approaches were applied to assess performance of the two regions in the identification of species but also any differences related to the curation of the corresponding databases; next, species substitutions and frauds detected in the shark food chain were linked to the European legislation on commercialization of threatened, endangered and/or prohibited species.

## 2. Materials and Methods

### 2.1. Samples Collection

A total of 130 samples of shark fillets, made up of 42 labeled as palombo (*Mustelus mustelus*, *Mustelus asterias, Mustelus punctulatus*), 35 as verdesca (*Prionace glauca*) 28 as gattuccio (*Scyliorhinus canicula*) and 25 as spinarolo (*Squalus acanthias, Squalus blainville*) (commercial and scientific names according to Italian MiPAAF Decree dated 22 September 2017) [52] were purchased from fish markets and fishmongers, in the Apulia region (south-eastern Italy), and stored at −20 °C until further use.

### 2.2. Fish Labeling Analysis

The mandatory labeling requirements indicated by Council Regulation (EC) No. 1379/2013 (Art. 35) were evaluated for each sample [53]. In detail, the commercial designation, the scientific name, the geographical area and production method, the category of fishing gear used in the capture of the species and whether previously frozen were assessed.

### 2.3. DNA Extraction and Purification

Genomic DNA extraction and purification were performed starting from 10 mg aliquots of sample, using the DNeasy Blood and Tissue Kit (QIAGEN, Hilden, Germany) as reported by Handy et al. [54]

Specimens of authentic species were used as positive extraction controls. No added tissue was included as negative extraction control to verify the purity of the extraction reagents. The DNA concentration and purity were established by evaluating the A260 nm/A280 nm ratio using a BioPhotometer D30 filter (Eppendorf, Milan, Italy).

### 2.4. Oligonucleotide Primers and Reference Genes

The universal primer set used in this study (Table 1), described by Ward et al. [55] and Naylor et al. [40] and synthesized by EUROFINS GENOMICS s.r.l. (Ebersberg, Germany), targeted the mitochondrial partial sequences of the cytochrome oxidase subunit I (COI) and NADH dehydrogenase subunit 2 (NADH2) mitochondrial DNA gene.

### 2.5. COI and NADH2 PCR Assay

The PCR reactions were conducted in a final volume of 25 μL, using 12.5 μL of HotStarTaq Master Mix 2× (QIAGEN, Hilden, Germany), containing 2.5 units of HotStarTaq DNA Polymerase, 1.5 mM of MgCl_2_ and 200 μL of each dNTP. Subsequently 0.5 μM of each primer and 1 μL of DNA were added. The amplification profiles for COI and NADH2 are described in Table 2.

Extraction and PCR positive and negative controls were encompassed. The PCR reactions were processed in an Applied Biosystems™ SimpliAmp™ Thermal Cycler (Thermo Fisher Scientific, Milan, Italy). All reactions were performed in duplicate.

### 2.6. Detection of Amplified Products

PCR amplified products were electrophoretically analyzed on 1.5% (*w*/*v*) agarose NA (Pharmacia, Uppsala, Sweden) gel in 1× Tris Acetate-EDTA buffer (TAE) containing 0.089 M Tris, 0.089 M acetic acid, 0.002 M EDTA, pH 8.0 (USB, Cleveland, OH, USA), and then stained with Green Gel Safe10,000× Nucleic Acid Stain (5 μL/100 mL) (Fisher Molecular Biology, Rome, Italy). A Gene Ruler^TM^ 100 bp DNA Ladder Plus (MBI Fermentas, Vilnius, Lithuania) was used as the molecular weight marker. Images were acquired using the Gel Doc™ EZ Imaging System with Image Lab™ Software (Bio-rad, Milan, Italy).

### 2.7. PCR Cleanup

The QIAquick PCR Purification Kit (QIAGEN, Hilden, Germany) was used to purify the PCR products, in order to generate an amplicon free of extra dNTPs and excess primers that might interfere with the sequencing reaction.

### 2.8. Cycle Sequencing Reaction

Sequencing reactions using forward and reverse COI and NADH2 universal primers were performed by EUROFINS GENOMICS s.r.l. (Ebersberg, Germany)

### 2.9. Analysis of Sequences

The sequences generated were dereplicated to identify and remove identical sequences using mothur v.1.44.1 [56]. Identification by analysis of similarity was performed, in the case of COI fragments, with a blast search against the Species Level Barcode Records database within BOLD SYSTEMS (http://www.boldsystems.org/index.php/IDS_OpenIdEngine) whereas the NADH2 fragments were blasted against the GenBank nucleotide database [57]. Summary of identifications of specimens based on analysis of similarity was visualized using Circos [58].

Identification by generation and placement in a tree was also performed. For COI analysis, all the reference sequences belonging to the species expected from the original label (*M. mustelus, M. asterias, M. punctulatus, Prionace glauca, Scyliorhinus canicula, Squalus acanthias* and *Squalus blainville*) and/or those not expected but recovered with a match <98% of similarity in the blast output, were downloaded from the BOLD SYSTEM. The reference sequences were dereplicated (i.e., identical sequences were removed) and aligned with the samples using MAFFT [59]. The alignment was manually checked and edited using SeaView v.4.0 [60].

A Maximum Likelihood tree was built with IQ-TREE v1.6.8 [61] with 1000 bootstrap replicates. The best-fit model was selected using ModelFinder [62] implemented in an IQ-TREE. The tree was visualized and annotated in iTOL [63,64]. An identical procedure was used for NADH2. For this marker, the hits were downloaded from the GenBank outputs, then dereplicated and multialigned with the samples using MAFFT to build the tree. *Chimaera monstrosa* was taken as outgroup both for COI (MN397913.1) and NADH2 (JQ518716.1) trees [65].

### 2.10. Assessment Conservation Status

The conservation status of each species identified was determined based on the IUCN Red List of Endangered Species [12], the appendices of CITES (Convention on International Trade of Endangered Species of Wild Fauna and Flora) [66], Barcelona Convention for the protection of the Mediterranean [67] and Bern Convention (Convention on the Conservation of European Wildlife and Natural Habitats) [68].

## 3. Results

### 3.1. Analysis of the Fish Labels

Despite being required by Article 35 of EU Regulation No 1379/2013 [53], none of the shark fillet labels showed complete information on commercial designations, scientific denominations, geographical origins, production methods, categories of fishing gear used in capture and whether they were previously frozen. The labels included only the Italian commercial designation (as per Italian MiPAAF Decree dated 22 September 2017) [52], while the scientific name, geographical area and category of fishing gear used in capture were missing from all samples.

### 3.2. Data Analysis

DNA of good yield and quality was isolated and purified from all 130 specimens. Following all of these extractions, the PCR products were clearly visible as single bands of the predicted size, i.e., respectively ~655 for COI and ~1050 for NADH2. Positive and negative controls for the extraction and PCR gave the predicted results. Sequence lengths ranged between 657–724 bp (average 694) in COI and 935–1391 bp (average 1084) in NADH2. After the removal of redundant haplotypes (fragments identical in length and nucleotide), the 130 samples were reduced to 20 COI and 22 NADH2 unique haplotypes (Table 3).

### 3.3. DNA-Based Species Identification

The clustering of haplotypes was in most cases congruent for both markers and contained samples originally labelled as the same species. However, in four cases in COI and three in NADH2, the cluster included a mix of specimens originally labelled as different species, highlighting the presence of mislabeling. Different numbers of total clusters in the two markers was due to two clusters including mixed specimens: (i) in COI, a cluster containing verdesca and spinarolo samples (*15C_Verd, 16C_Verd, 25C_Verd, 33C_Spin*) was split into two clusters in NADH2, one including verdesca and spinarolo *(15N_Verd, 33N_Spin*) and another only with verdesca (*16N_Verd, 25N_Verd)*; (ii) in COI, a cluster containing verdesca and spinarolo samples (*17C_Verd, 3C_Spin)* was split into two *3N_Spin* and *17N_Verd* (Table 3). Analysis of similarity gave successful matches with references available, with the pair-wise sequence identity fluctuating from 99% to 100%.

**Figure 1:**
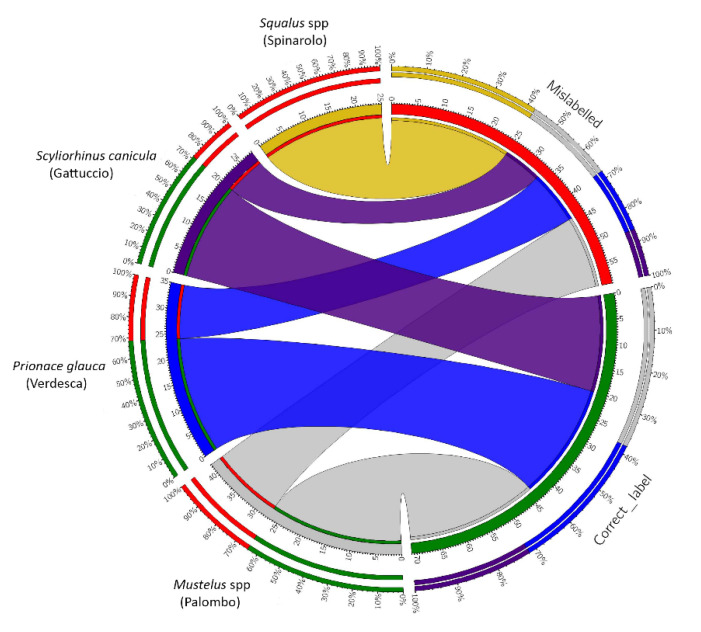
Circos plot summarizing correspondence between the original label of samples and the molecular identification by similarity. On the left, results are reported based on species. Bundle colors represent the four species expected from the original labels (Palombo gray; Verdesca blue; Gattuccio purple; Spinarolo gold). Within each species, the bundle is split into two parts with width corresponding to the relative abundance of samples found to be correctly labelled or mislabeled. Only in spinarolo does the presence of a whole bundle highlight the absence of correct matches. On the right, results are reported based on identifications (correct or mislabeled). The inner ring reports the total number of specimens with correct labels (green) and mislabeled (red), while the external parts show the relative abundance of each species for each condition (correct or incorrect label) using the color of the species.

The results of molecular investigations for both markers revealed a high occurrence of incorrect species declaration in 59/130 (45.4%) shark meat products. Of these, 15/42 (35.7%) fillets of palombo (*M. mustelus, M. asterias, M. punctulatus*) were wrongly labeled, with 9/15 being identified as *Scyliorinus canicula* and 6/15 as *Prionace glauca*. Among the prepared fillets sold as verdesca (*Prionace glauca*), 11/35 (31.4%) were incorrectly labeled, with 6/11 samples identified as belonging to *Mustelus asterias* and 5/11 as *Isurus oxyrinchus*. In addition, post-sequencing data analysis found that 8/28 (28.6%) fillets of gattuccio (*Scyliorhinus canicula*) were not correctly labeled, with 8/8 being identified as belonging to *Mustelus punctulatus.* Finally, all purported spinarolo (*Squalus acanthias, Squalus blainville*) (100%) samples were mislabeled, all being identified as *Prionace glauca*. Details of blast results are reported in Figure 1 and Supplementary Material Tables S1–S4.

**Figure 2:**
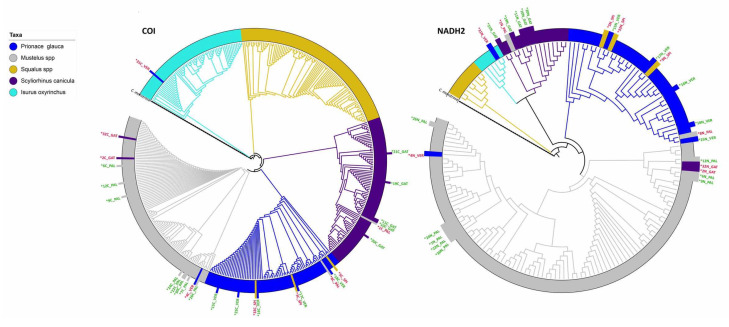
Maximum likelihood trees for the two markers including the reference sequences, outgroup (black dash line) and the specimens to identify. Leaves corresponding to the specimens (taller bars) have the color corresponding to that of the original labels while the names are colored based on the congruence (green) or incongruence (red) between the original label and tree placement.

The final alignment of COI produced a matrix of 424 sequences with 590 characters with best-fit model HKY + F + I + G4 chosen according to BIC criterion. In NADH2, the final alignment produced a matrix of 163 sequences with 865 characters with best-fit model TIM2 + F + I + G4 according to BIC criterion. In the COI and NADH2 Maximum likelihood trees (Figure 2), the placement of samples confirmed the identification (correctly or incorrectly labeled samples) obtained by the similarity approach.

The list of references used for the generation of the COI and NADH2 trees is available in Supplementary Material Tables S5–S7.

### 3.4. Conservation Status of Identified Species

The study highlighted the use of threatened species in the International Union for Conservation of Nature (IUCN) in the Mediterranean (critically endangered-CR): *Prionace glauca* and *Isurus oxyrinchus* [12] (Table 4). None of the species was associated with CITES listings, prohibitions on landings or restricted sales [66]. The study detected the use of species included in Annex II (List of endangered and threatened species) of the Barcelona Convention for the Protection of the Mediterranean [67], such as *Isurus oxyrinchus* and species listed in Appendix III (Protected Fauna Species) of the Bern Convention such as *Isurus oxyrinchus* and *Prionace glauca* [68] (Table 4).

## 4. Discussion

The present study represents further evidence that mislabeling of seafood products, especially of processed and prepared seafood, occurs on a global scale, confirming previous studies and reports which provide documentary evidence that fish fraud through mislabeling and species substitution is a widespread issue for fish and fishery products [7,9,10].

This study firstly shows that the labeling of shark meat products often completely lacks the scientific name, showing only the common or local denomination, which makes it difficult for consumers to make informed choices and fails to follow EU legislation regarding labelling of seafood. Indeed, EU Reg. 1379/2013 [53] established strict legislation governing seafood labeling and the provision of key information to consumers, such as commercial and scientific names, geographical area, production method, category of fishing gear used in capture, as well as whether the product has been previously frozen, thus ensuring their traceability and identification throughout the supply chain.

Molecular investigation detects a high occurrence of incorrect species declaration in shark meat products in agreement with other studies [7,9,69]. The high mislabeling rate (45.4%) observed in this study is in line with the Oceania Report, which showed that 33% of seafood from retail outlets in the United States was mislabeled [70], and Marko et al. [8], who reported that between 60% and 94% of fish sold as red snapper did not comply with their label. Barbuto et al. [71], Filonzi et al. [10] and Armani et al. [72] found that 80%, 32% and 48.5% respectively of analyzed samples in Italy revealed an incorrect fish species declaration. Moreover, a high rate of commercial fraud, of about 60%, in shark species was observed by Giovos et al. [6] and Pazartzi et al. [7].

This study highlighted widespread use of cheaper species, such as *Scyliorhinus canicula* and *Prionace glauca*, to replace species that are better known and appreciated by Italian consumers, such as *M. mustelus; M. asterias;* and *M. punctulatus* among shark meat products. Further, among samples labelled as spinarolo, this investigation showed that *Squalus acanthias* and *Squalus blainville*, species commonly found on Italian coasts and which have the most prized meats, were replaced with *Prionace glauca.* On the other hand, the study shows that incorrectly labeled fillets sold as gattuccio (*Scyliorhinus canicula*) and as verdesca (*Prionace glauca*) were identified as belonging to *Mustelus punctulatus* and *Mustelus asterias,* respectively. Therefore, these results would argue that the high replacement rate observed may be encouraged by difficulties in identifying morphologically similar species, especially among prepared shark meat products. Taxonomically speaking, removing head and fins makes it more challenging to identify species reliably based on morphological traits, thus allowing shark carcasses to be sold fraudulently [73]. Therefore, the similar high variety of fillets or fillets of commercialized shark species and the overall lack of taxonomical expertise, in addition to depletion in the Mediterranean Sea of the best known and most appreciated species [15], make it hard to mitigate the negative effects of human shark consumption and to inform consumers as to whether products come from a threatened species or have been illegally traded.

Indeed, analysis of prepared shark meat products revealed marketing of different threatened species, including endangered and Barcelona and Bern Convention-listed ones, thus confirming that seafood mislabeling can prejudice sustainable fisheries management and facilitate Illegal, Unreported and Unregulated (IUU) fishing [74]. Specifically, among the samples of verdesca, the results also highlight significant sustainability issues regarding the use of *Isurus oxyrinchus*, a species classified as Endangered (EN) due to a severe population decline [12] and listed in Appendix II of the Barcelona Convention among endangered and threatened species [67] and in Appendix III of the Bern Convention among protected fauna species [68]. In addition, its capture by bottom nets is prohibited in the Mediterranean according to Regulations 2006/1967 and 2019/1241 [75,76]. The use of *Isurus oxyrinchus* to replace *Prionace glauca* in this study could be from unreported and unregulated illegal (IUU) fishing landed at Italian ports. In fact, mislabeling of fish products is used either to launder IUU fish into the legal marketplace or else simply to defraud the industry and consumer in order to garner a higher sale price [5,9]. Further, among the samples labeled as palombo, the over-exploitation in the Mediterranean Sea of *M. mustelus,* currently classified as vulnerable on the IUCN Red List [12], could justify the species’ non-identification [15,76]. The replacement of *Squalus acanthias* and *Squalus blainville* could also be attributed to their decrease in the Mediterranean Sea as overfished species, classified as endangered and data-deficient respectively in the IUCN Red List [12,15]. In addition, in 2019, EU Regulation 124/2019 (Articles 14, 34, 50) [77] prohibited the fishing, storage on board, trans-shipment and landing of *Squalus acanthias*.

Considering the increasing decline worldwide of shark species, this study highlights the need to manage shark populations sustainably, given that using Endangered (EN), Vulnerable (VU) or Near-Threatened (NT) species may contribute to their extinction. A huge threat for the sustainable management of these valuable resources is unregulated shark meat landings and commercialization [78]. Interestingly, the widespread use of *Prionace glauca* to replace spinarolo and palombo, in this study, could highlight significant conservation implications, as it is another species in serious decline, classified as Near-Threatened on the IUCN Red List [12] and listed in Appendix III of the Bern Convention [68] among protected fauna species, especially within the Mediterranean, where population trends for many shark species are negative because of over-exploitation [15]. On the other hand, it is worthwhile underlining that the use of *Prionace glauca* may well indicate intentional and fraudulent deception of consumers, as it is a larger species with no morphological similarity to *Mustelus* spp., *Squalus acanthias* and *Squalus blainville* [7].

In addition to conservation and commercial concerns, this study also gives rise to food safety and consumer health concerns. Given their position at the head of the food chain, sharks are particularly prone to bioaccumulate toxic metals, suggesting that their consumption may be consumers’ main source of exposure to lead, cadmium, arsenic and mercury and that there are likely to be potential health risks due to long-term exposure. Indeed, mercury levels in some larger shark species such as *Prionace glauca* and *Isurus oxyrinchus*, have been found to be up to four times higher than the legal maximum [79,80]. Further, Mull et al. [81] observed that high contamination levels in sharks are directly connected with geographical areas that contained high levels of pollutants. Therefore, the use of such ambiguous labels with species information completely missing in markets and fishmongers greatly hampers consumers from exercising their right to avoid species associated with specific health issues [82,83,84].

From a legislative point of view, this study underlines the need for rigorous application of Regulation (EU) No 1379/2013 [53], not only to protect consumers against fraud, but also for its role in preventing IUU and conserving vulnerable and endangered sharks, which will otherwise continue to be sold to consumers in Italy, with huge effects on biodiversity and on the conservation of this species [85]. It is essential to harmonize the commercial designation of fish in the EU to train professional fishermen and competent authorities in the identification of vulnerable and protected shark species and in order to implement authenticity assessments in the fishery sector and thus to combat mislabeling and illegal fishing. This study represents further evidence that a comprehensive traceability system is needed for seafood products as set out in Reg EC 178/2002 [86]. If the implementation and application of a strict traceability process within the fisheries supply chain were to be recommended by European legislation, this would allow a complete control system to be set up, ensuring that all consignments of fishery and aquaculture products can be traced at all stages of production, processing and distribution, from capture or harvesting to retail (Regulation EC 1224/2009, Article 58) [87]. The need to improve traceability and transparency in the fishing sector by implementing full traceability is a crucial step in eliminating illegal fishing, seafood mislabeling and fraud [9]. In order to contrast fishery fraud, the implementation of a reliable traceability system that would track fish from the point of harvest to the consumer’s plate is required.

Contemporary traceability systems are based on a paper trail that traces various data including geographical origin, species and vessel registration details. Experience from previous cases of food fraud (e.g., the horsemeat scandal that occurred in Europe) demonstrates that such documentation may be falsified. Moreover, fish sellers should be subject to stricter and more regular controls, as indicated in the new Regulation (EU) 625/2017 [88] on official controls, thus preventing products of dubious or unknown origin from reaching consumers, as well as discouraging illegal fishing practices.

Prevention of species substitution and mitigation of the practice of generalized and nonspecific labeling in fish markets require the availability of fast, cheap, reliable and potentially automated methods. The European Parliament report on food crises and fraud [89] proposed DNA-based methods as the standard strategy to identify fish species and for surveillance of commercial fraud throughout the production chain up to the final delivery of fish products to consumers. Analysis of the mitochondrial genome, by using DNA barcoding, offers a powerful system for the identification of species even when specimens are either incomplete or belong to species that exhibit cryptic diversity. Specifically, the DNA sequencing used in this study is a helpful tool for the authentication of shark species in fresh fish and lightly-processed products with total reliability, and adequate analysis costs in less than two working days, permitting high-throughput screening of all kinds of shark samples. Nevertheless, the manufacturing processes, such as high temperature, high pressure and the addition of certain ingredients, used by the food industry, may cause DNA degradation, that may affect the quantity and quality of DNA extracted from these products, precluding the use of PCR amplification of full-length (i.e., ~ 650 bp) barcodes [90]. In order to obtain DNA sequence information from commercially processed fishery products with degraded DNA, a mini-barcoding approach, which analyses shorter DNA fragments (e.g., 100–200 bp) could be efficient and effective for species identification [91].

In this study, a multi-marker DNA barcoding approach provided reliable and accurate discrimination of shark samples. In our dataset, the two mitochondrial regions COI and NADH2 reveal the same capability to uncover food fraud, showing agreement in the identification of samples using analysis by both similarity and tree placement. Although the two markers display the same performances in our dataset, the use of COI provides more straightforward identification given the availability of the BOLD curated resource. Indeed, even though the BOLD database also includes sequences mined from GenBank, molecular reference data are harmonized by length and the blast search provides results organized in such a way as to make it easy to identify down to species or genus level. By contrast, for NADH2 fragments, identification against the GenBank nucleotide database provided different best hits where results were sorted by query coverage, percentage identity or total score. The discrepancy, due to the BLAST algorithm that attempts to extend the match both forwards and backwards, continuing the extension as long as the alignment score continues to increase, could generate pitfalls in the correct reading and interpretation of the output of blast in cases of sister species or an absence of target species. Considering that all the resources available for identification (BOLD included) are tools created for the exploration of biodiversity data, more efforts are required to organize online databases dedicated to targeting seafood frauds, in order to implement more rigorous food control programs [34]. Results of the performance of the two markers and patterns on fraudulent activities will be important for future studies targeting traceability in the fisheries sector, managing seafood safety, environmental sustainability and food product authenticity. This methodology might also be useful to verify imports and exports of these species and to determine their geographical origin. This would enable the allocated total allowable catches (TACs) for these species to be checked, as well as helping to prevent Illegal, Unreported and Unregulated (IUU) fishing practices [74]. 

Moreover, the study indicates that the routine use of genetic analysis should be encouraged in control and enforcement agencies. Therefore, DNA-analysis techniques in fisheries and aquaculture compliance investigations could make it possible to control shark fisheries, implementing efficient management measures, establishing a species-specific reporting system for all catches, and strengthening control measures, in order to prevent illegal activities connected with shark catches and trade around the world [92,93,94,95].

## Figures and Tables

**Table 1:** Oligonucleotide primers—target genes.

Target Gene	Primer Sequences (5′–3′)	Amplicon Length (bp)	References
COI	F- TCAACCAACCACAAAGACATTGGCAC	~655	[55]
	R- TAGACTTCTGGGTGGCCAAAGAATCA		
NADH2	F- AAGGAGCAGTTTGATAGAGT	~1050	[40]
	R- AACGCTTAGCTGTTAATTAA		

**Table 2:** COI and NADH2 amplification profiles.

Target Gene	Heat Activation	Denaturation	Annealing	Extension	Cycles	Final Extension
COI	95 °C/15 min	94 °C/30 s	54 °C/50 s	72 °C/60 s	35	72 °C/10 min
NADH2	95 °C /15 min	94 °C/30 s	48 °C/30 s	72 °C/90 s	35	72 °C/10 min

**Table 3:** Summary of groups of identical haplotypes for COI and NADH2 markers. Groups of haplotypes including samples originally labelled as different species are indicated in bold.

Haplotype	COI	NADH2
*H1*	1C_Pal (9 specimens)	1N_Pal (9 specimens)
*H2*	6C_Pal (4 specimens), 9C_Pal (4 specimens)	6N_Pal (4 specimens), 9N_Pal (4 specimens)
*H3*	7C_Pal (3 specimens)	7N_Pal (3 specimens)
*H4*	**8C_Pal (6 specimens), 18C_Verd (5 specimens)**	**8N_Pal (6 specimens), 18N_Verd (5 specimens)**
*H5*	**12C_Pal (4 specimens), 32C_Gat (4 specimens)**	**12N_Pal (4 specimens), 32N_Gat (4 specimens)**
*H6*	22C_Pal (3 specimens)	22N_Pal (3 specimens)
*H7*	24C_Pal (3 specimens)	24N_Pal (3 specimens)
*H8*	26C_Pal (3 specimens)	26N_Pal (3 specimens)
*H9*	30C_Pal (3 specimens)	30N_Pal (3 specimens)
*H10*	4C_Verd (6 specimens)	4N_Verd (6 specimens)
*H11*	**15C_Verd (5 specimens), 16C_Verd (5 specimens), 25C_Verd (4 specimens), 33C_Spin (7 specimens)**	**15N_Verd (5 specimens),** **33N_Spin (7 specimens)**
*H12*	-	16N_Verd (5 specimens), 25N_Verd (4 specimens)
*H13*	**17C_Verd (5 specimens), 3C_Spin (7 specimens)**	17N_Verd (5 specimens)
*H14*	-	3N_Spin (7 specimens)
*H15*	31C_Verd (5 specimens)	31N_Verd (5 specimens)
*H16*	2C_Gat (4specimens)	2N_Gat (4 specimens)
*H17*	10C_Gat (4 specimens)	10N_Gat (4 specimens)
*H18*	11C_Gat (4 specimens)	11N_Gat (4 specimens)
*H19*	19C_Gat (4 specimens)	19N_Gat (4 specimens)
*H20*	20C_Gat (4specimens)	20N_Gat (4 specimens)
*H21*	21C_Gat (4 specimens)	21N_Gat (4 specimens)
*H22*	5C_Spin (11 specimens)	5N_Spin (11 specimens)

**Table 4:** Conservation status of identified species.

Species	Number of Samples	Conservation Status			
		IUCN *	CITES	Barcelona Convention **	Bern Convention
*Mustelus asterias*	21	LC	--	Annex III	--
*Mustelus punctulatus*	20	DD	--	Annex III	--
*Prionace glauca*	55	CR	--	Annex III	Appendix III
*Isurus oxyrinchus*	5	CR	--	Annex II	Appendix III
*Scyliorhinus canicula*	29	LC	--	--	--

* IUCN (International Union for the Conservation of Nature) Conservation status categories: Least Concern (LC), Data-deficient (DD); Near-Threatened (NT), Endangered (EN). CITES: Convention on International Trade of Endangered Species of Wild Fauna and Flora ** Barcelona Convention: Annex II: List of endangered and threatened species; Annex III: List of species whose exploitation is regulated.

## Supplementary Materials

The following are available online at https://www.mdpi.com/2304-8158/9/9/1194/s1, Table S1: Blast Results “Palombo” fillets, Table S2: Blast Results “Verdesca” fillets, Table S3: Blast Results “Gattuccio” fillets, TableS4: Blast Results “Spinarolo” fillets, Table S5: References included in COI tree downloaded from BOLD but mined from GenBank, Table S6: References included in COI tree from BOLD, Table S7: References included in NADH2 tree downloaded from GenBank.

## References

[1] Hellberg R.S., Isaacs R.B., Hernandez E.L. (2019). Identification of shark species in commercial products using DNA barcoding. Fish. Res..

[2] Bradai M.N., Saidi B., Enajjar S. (2012). Elasmobranchs of the Mediterranean and Black Sea: Status, Ecology and Biology Bibliographic Analysis.

[3] Clarke S. (2004). Shark Product Trade in Hong Kong and Mainland China and Implementation of the CITES Shark Listings.

[4] Dent F., Clarke S. (2015). State of the Global Market for Shark Products.

[5] Hobbs C.A.D., Potts R.W.A., Walsh M.B., Usher J., Griffiths A.M. (2019). Using DNA Barcoding to investigate patterns of species utilisation in UK shark products reveals threatened species on sale. Sci. Rep..

[6] Giovos I., Arculeo M., Doumpas N., Katsada D., Maximiadi M., Mitsou E., Paravas V., Aga-Spyridopoulou R.N., Stoilas V.O., Tiralongo F. (2020). Assessing multiple sources of data to detect illegal fishing, trade and mislabeling of elasmobranchs in Greek markets. Mar. Policy.

[7] Pazartzi T., Siaperopoulou S., Gubili C., Maradidou S., Loukovitis D., Chatzispyro A., Griffiths A.M., Minos G., Imsiridou A. (2019). High levels of mislabeling in shark meat—Investigating patterns of species utilization with DNA barcoding in Greek retailers. Food Control..

[8] Marko P.B., Lee S.C., Rice A.M., Gramling J.M., Fitzhenry T.M., McAlister J.S., Harper G.R., Moran A.L. (2004). Mislabelling of a depleted reef fish. Nature.

[9] Di Pinto A., Marchetti P., Mottola A., Bozzo G., Bonerba E., Ceci E., Bottaro M., Tantillo G. (2015). Species identification in fish fillet products using DNA barcoding. Fish. Res..

[10] Filonzi L., Chiesa S., Vaghi M., Marzano F.N. (2010). Molecular barcoding reveals mislabelling of commercial fish products in Italy. Food Res. Int..

[11] Miller D., Jessel A., Mariani S. (2012). Seafood mislabeling: Comparisons of two western European case studies assist in defining influencing factors, mechanisms and motives. Fish. Fish..

[12] IUCN The IUCN Red List of Threatened Species. Version 2020.1. www.iucnredlist.org.

[13] Carr L.A., Stier A.C., Fietz K., Montero I., Gallagher A.J., Bruno J.F. (2013). Illegal shark fishing in the Galápagos Marine Reserve. Mar. Policy.

[14] European Commission (2018). The EU Food Fraud Network and the System for Administrative Assistance—Food Fraud.

[15] WWF (2019). Sharks in Crisis: A Call to Action for the Mediterranean.

[16] Wilkens H., Strecker U. (2003). Convergent evolution of the cavefish Astyanax (Characidae, Teleostei): Genetic evidence from reduced eye-size and pigmentation. Biol. J. Linn. Soc..

[17] Li X., Shen X., Chen X., Xiang D., Murphy R.W., Shen Y. (2018). Detection of potential problematic *Cytb* gene sequences of fishes in GenBank. Front. Genet..

[18] Hebert P.D.N., Ratnasingham S., de Waard J.R. (2003). Barcoding animal life: Cytochrome c oxidase subunit 1 divergences among closely related species. Proc. R. Soc. B Biol. Sci..

[19] Ratnasingham S., Hebert P.D. (2007). bold: The barcode of life data system (http://www.barcodinglife.org). Mol. Ecol. Notes.

[20] FishBase. www.fishbase.org.

[21] Ward R.D. (2012). FISH-BOL, a case study for DNA barcodes. Methods Mol. Biol..

[22] Becker S., Hanner R., Steinke D. (2011). Five years of FISH-BOL: Brief status report. Mitochondr. DNA.

[23] Steinke D., Hanner R. (2011). FISH-BOL. The FISH-BOL collaborators’ protocol. Mitochondr. DNA.

[24] Nneji L.M., Adeola A.C., Mustapha M.K., Oladipo S.O., Djagoun C.A., Nneji I.C., Adedeji B.E., Olatunde O., Ayoola A.O., Okeyoyin A.O. (2020). DNA barcoding silver butter catfish (Schilbe intermedius) reveals patterns of mitochondrial genetic diversity across African river systems. Sci. Rep..

[25] Fadli N., Nor S.A.M., Othman A.S., Sofyan H., Muchlisin Z.A. (2020). DNA barcoding of commercially important reef fishes in Weh Island, Aceh, Indonesia. PeerJ.

[26] Arroyave J., Martinez C.M., Stiassny M.L. (2019). DNA barcoding uncovers extensive cryptic diversity in the African long-fin tetra *Bryconalestes longipinnis* (Alestidae: Characiformes). J. Fish. Biol..

[27] Escobar R., Luna-Acosta A., Caballero S. (2020). DNA barcoding, fisheries and communities: What do we have? Science and local knowledge to improve resource management in partnership with communities in the Colombian Caribbean. Mar. Policy.

[28] Galal-Khallaf A., Ardura A., Mohammed-Geba K., Borrell Y.J., Garcia-Vazquez E. (2014). DNA barcoding reveals a high level of mislabeling in Egyptian fish fillets. Food Control.

[29] Hu Y., Huang S.Y., Hanner R., Levin J., Lu X. (2018). Study of fish products in Metro Vancouver using DNA barcoding methods reveals fraudulent labeling. Food Control.

[30] Barendse J., Roel A., Longo C., Andriessen L., Webster L.M., Ogden R., Neat F. (2019). DNA barcoding validates species labelling of certified seafood. Curr. Biol..

[31] FDA. https://www.fda.gov/food/dna-based-seafood-identification/single-laboratory-validated-method-dna-barcoding-species-identification-fish.

[32] Wong E.H.K., Shivji M.S., Hanner R.H. (2009). Identifying sharks with DNA barcodes: Assessing the utility of a nucleotide diagnostic approach. Mol. Ecol. Res..

[33] Bucklin A., Steinke D., Blanco-Bercial L. (2011). DNA barcoding of marine metazoa. Annu. Rev. Mar. Sci..

[34] Zanzi A., Martinsohn J.T. (2017). FishTrace: A genetic catalogue of European fishes. Database.

[35] Jeon H.B., Anderson D., Won H., Lim H., Suk H.Y. (2018). Taxonomic characterization of Tanakia species (*Acheilognathidae*) using DNA barcoding analyses. Mitochondr. DNA.

[36] Horreo J.L., Fitze P.S., Jiménez-Valverde A., Noriega J.A., Pelaez M.L. (2019). Amplification of 16S rDNA reveals important fish mislabeling in Madrid restaurants. Food Control.

[37] Valentini A., Taberlet P., Miaud C., Civade R., Herder J., Thomsen P.F., Bellemain E., Besnard A., Coissac E., Boyer F. (2016). Next-generation monitoring of aquatic biodiversity using environmental DNA metabarcoding. Mol. Ecol..

[38] Collins R.A., Bakker J., Wangensteen O.S., Soto A.Z., Corrigan L., Sims D.W., Genner M.J., Mariani S. (2019). Non-specific amplification compromises environmental DNA metabarcoding with COI. Methods Ecol. Evol..

[39] Moore A.B.M., White W.T., Ward R.D., Naylor G.J.P., Peirce R. (2011). Rediscovery and redescription of the smoothtooth blacktip shark, Carcharhinus leiodon (Carcharhinidae), from Kuwait, with notes on its possible conservation status. Mar. Freshw. Res..

[40] Naylor G.J.P., Caira J.N., Ensen K.J., Rosana K.A.M., White W.T., Last P.R. (2012). A DNA sequence–based approach to the identification of shark and ray species and its implications for global elasmobranch diversity and parasitology. Bull. Am. Mus. Nat. Hist..

[41] Henderson A.C., Reeve A.J., Jabado R.W., Naylor G.J.P. (2016). Taxonomic assessment of sharks, rays and guitarfishes (Chondrichthyes: Elasmobranchii) from south-eastern Arabia, using the NADH dehydrogenase subunit 2 (NADH2) gene. Zool. J. Linn. Soc..

[42] Last P.R., Seret B., Naylor G.J. (2016). A new species of guitarfish, *Rhinobatos borneensis* sp. nov. with a redefinition of the family-level classification in the order *Rhinopristiformes* (Chondrichthyes: Batoidea). Zootaxa.

[43] Vella A., Vella N., Schembri S. (2017). A molecular approach towards taxonomic identification of elasmobranch species from Maltese fisheries landings. Mar. Genom..

[44] Feitosa L.M., Martins A.P.B., Giarrizzo T., Macedo W., Monteiro I.L., Gemaque R., Nunes J.L.S., Gomes F., Schneider H., Sampaio I. (2018). DNA-based identification reveals illegal trade of threatened shark species in a global elasmobranch conservation hotspot. Sci. Rep..

[45] Cutarelli A., Amoroso M.G., De Roma A., Girardi S., Galiero G., Guarino A., Corrado F. (2014). Italian market fish species identification and commercial frauds revealing by DNA sequencing. Food Control..

[46] Guardone L., Tinacci L., Costanzo F., Azzarelli D., D’Amico P., Tasselli G., Magni A., Guidi A., Nucera D., Armani A. (2017). DNA barcoding as a tool for detecting mislabeling on incoming fishery products from third countries: An official survey conducted at the Border Inspection Post of Livorno-Pisa (Italy). Food Control.

[47] Sarmiento K.P., Pereda J.M.R., Ventolero M.F.H., Santos M.D. (2018). Not fish in fish balls: Fraud in some processed seafood products detected by using DNA barcoding. Phil. Sci. Lett..

[48] Zeng L., Wen J., Fan S., Chen Z., Xu Y., Sun Y., Chen D., Zhao J. (2018). Species identification of fish maw (Porcupinefish) products sold on the market using DNA sequencing of 16S rRNA and COI genes. Food Control..

[49] Acutis P.L., Cambiotti V., Riina M.V., Meistro S., Maurella C., Massaro M., Stacchini P., Gili S., Malandra R., Pezzolato M. (2019). Detection of fish species substitution frauds in Italy: A targeted National Monitoring Plan. Food Control.

[50] Deconinck D., Volckaert F.A., Hostens K., Panicz R., Eljasik P., Faria M., Monteirod C.S., Robbens J., Derycke S. (2020). A high-quality genetic reference database for European commercial fishes reveals substitution fraud of processed Atlantic cod (*Gadus morhua*) and common sole (*Solea solea*) at different steps in the Belgian supply chain. Food Chem. Toxicol..

[51] Kolmann M.A., Elbassiouny A.A., Liverpool E.A., Lovejoy N.R. (2017). DNA barcoding reveals the diversity of sharks in Guyana coastal markets. Neotrop. Ichthyol..

[52] (2017). Ministerial Decree n°19105 of 22 September 2017. Italian Name for Fish Species of Commercial Interest -Annex 1.

[53] (2013). Regulation (EU) No 1379/2013 of the European Parliament and of the Council of 11 December 2013on the common organization of the markets in fishery and aquaculture products, amending Council Regulations (EC) No 1184/2006 and (EC) No 1224/2009 and repealing Council Regulation (EC) No 104/2000. Off. J. EU.

[54] Handy S.M., Deeds J.R., Ivanova N.V., Hebert P.D.N., Hanner R.H., Ormos A., Weigt L.A., Moore M.M., Yancy H.F. (2011). A single-laboratory validated method for the generation of DNA barcodes for the identification of fish for regulatory compliance. J. AOAC Int..

[55] Ward R.D., Zemlak T.S., Innes B.H., Last P.R., Hebert P.D.N. (2005). DNA barcoding Australia’s fish species. Philos. Trans. R. Soc. Lond. B..

[56] Schloss P.D., Westcott S.L., Ryabin T., Hall J.R., Hartmann M., Hollister E.B., Lesniewski R.A., Oakley B.B., Parks D.H., Robinson C.J. (2009). Introducing mothur: Open-source, platform-independent, community-supported software for describing and comparing microbial communities. Appl. Environ. Microbiol..

[57] GenBank Nucleotide Database. https://www.ncbi.nlm.nih.gov.

[58] Krzywinski M., Schein J., Birol I., Connors J., Gascoyne R., Horsman D., Marra M.A. (2009). Circos: An information aesthetic for comparative genomics. Genome Res..

[59] Katoh K., Standley D.M. (2013). MAFFT multiple sequence alignment software version 7: Improvements in performance and usability. Mol. Biol. Evol..

[60] Gouy M., Guindon S., Gascuel O. (2010). SeaView version 4: A multiplatform graphical user interface for sequence alignment and phylogenetic tree building. Mol. Biol. Evol..

[61] Minh B.Q., Schmidt H.A., Chernomor O., Schrempf D., Woodhams M.D., von Haeseler A., Lanfear R. (2020). IQ-TREE 2: New models and efficient methods for phylogenetic inference in the genomic era. Mol. Biol. Evol..

[62] Kalyaanamoorthy S., Minh B.Q., Wong T., von Haeseler A., Jermiin L. (2017). ModelFinder: Fast model selection for accurate Phylogenetic Estimates. Nat. Methods.

[63] iTOL. www.itol.embl.de/.

[64] Letunic I., Bork P. (2019). Interactive Tree of Life (iTOL) v4: Recent updates and new developments. Nucleic Acids Res..

[65] Pavan-Kumar A., Gireesh-Babu P., Babu P.S., Jaiswar A.K., Krishna V.H., Prasasd K.P., Chaudhari A., Raje S.G., Chakraborty S.K., Krishna G. (2014). Molecular phylogeny of elasmobranchs inferred from mitochondrial and nuclear markers. Mol. Biol. Rep..

[66] CITES APPENDIX. https://cites.org/eng/app/index.php.

[67] Barcelona Convention for the Protection of the Mediterranean. https://planbleu.org/sites/default/files/upload/files/Barcelona_convention_and_protocols_2005_eng.pdf.

[68] Bern Convention (Convention on the Conservation of European Wildlife and Natural Habitats). https://www.coe.int/en/web/bern-convention/appendices.

[69] Marino I.A.M., Riginella E., Cariani A., Tinti F., Farrell E.D., Mazzoldi C., Zane L. (2015). New molecular tools for the identification of 2 endangered smooth-hound sharks, *Mustelus mustelus* and *Mustelus Punctulatus*. J. Hered..

[70] Oceana Protecting the World’s Oceans: Annu. Report. 2018–2019. https://oceana.org/sites/default/files/oceana_annual_report_2018-2019_website.pdf.

[71] Barbuto M., Galimberti A., Ferri E., Labra M., Malandra R., Galli P., Casiraghi M. (2010). DNA barcoding reveals fraudulent substitutions in shark seafood products: The Italian case of “palombo” (*Mustelus* spp.). Food Res. Int..

[72] Armani A., Guardone L., Castellana R.L., Gianfaldoni D., Guidi A., Castigliego L. (2015). DNA barcoding reveals commercial and health issues in ethnic seafood sold on the Italian market. Food Control..

[73] Holmes B.H., Steinke D., Ward R.D. (2009). Identification of shark and ray fins using DNA barcoding. Fish. Res..

[74] Muttaqin E., Abdullah A., Nurilmala M., Ichsan M., Simeone B.M., Yulianto I., Booth H. (2019). DNA-barcoding as molecular marker for seafood forensics: Species identification of locally consumed shark fish products in the world’s largest shark fishery. Earth Environ. Sci..

[75] (2006). Council Regulation (EC) No 1967/2006 of 21 December 2006 concerning management measures for the sustainable exploitation of fishery resources in the Mediterranean Sea, amending Regulation (EEC) No 2847/93 and repealing Regulation (EC) No 1626/94. Off. J. EU.

[76] (2019). Regulation (EU) 2019/1241 of the European Parliament and of the Council of 20 June 2019 on the conservation of fisheries resources and the protection of marine ecosystems through technical measures, amending Council Regulations (EC) No 1967/2006, (EC) No 1224/2009 and Regulations (EU) No 1380/2013, (EU) 2016/1139, (EU) 2018/973, (EU) 2019/472 and (EU) 2019/1022 of the European Parliament and of the Council, and repealing Council Regulations (EC) No 894/97, (EC) No 850/98, (EC) No 2549/2000, (EC) No 254/2002, (EC) No 812/2004 and (EC) No 2187/2005. Off. J. EU.

[77] (2019). Council Regulation (EU) 2019/124 of 30 January 2019 fixing for 2019 the fishing opportunities for certain fish stocks and groups of fish stocks, applicable in Union waters and, for Union fishing vessels, in certain non-Union waters. Off. J. EU.

[78] FAO (2018). The State of World Fisheries and Aquaculture—Meeting Sustainable Development Goals.

[79] Maz-Courrau A., López-Vera C., Galván-Magaña F., Escobar-Sánchez O., Rosíles-Martínez R., Sanjuán-Muñoz A. (2012). Bioaccumulation and biomagnifications of total mercury in four exploited shark species in the Baja California Penisula, Maexico. Bull. Environ. Contam. Toxicol..

[80] RASFF (2018). The Rapid Alert System for Food and Feed. Annual Report 2018.

[81] Mull C.G., Lyons K., Blasius M.E., Winkler C., O’Sullivan J.B., Lowe C.G. (2013). Evidence of maternal offloading of organic contaminants in white sharks (*Carcharodon carcharias*). PLoS ONE.

[82] Castro-González M.I., Méndez-Armenta M. (2008). Heavy metals: Implications associated to fish consumption. Environ. Toxicol. Pharm..

[83] Karami A., Golieskardi A., Ho Y.B. (2017). Microplastics in eviscerated flesh and excised organs of dried fish. Sci. Rep..

[84] Williams M., Hernandez-Jover M., Shamsi S. (2020). Fish substitutions which may increase human health risks from zoonotic seafood borne parasites: A review. Food Control..

[85] Sadovy de Mitcheson Y., Andersson A.A., Hofford A., Law C.S.W., Hau L.C.Y., Pauly D. (2018). Out of control means off the menu: The case for ceasing consumption of luxury products from highly vulnerable species when international trade cannot be adequately controlled; shark fin as a case study. Mar. Policy.

[86] (2002). Regulation (EC) No. 178/2002 Of the European Parliament and of the Council of 28 January 2002 laying down the general principles and requirements of food law, establishing the European Food Safety Authority and laying down procedures in matters of food saf. Off. J. Eur. Commun..

[87] (2009). Regulation (EC) No 1224/2009 of 20 November 2009 establishing a Community control system for ensuring compliance with the rules of the common fisheries policy, amending Regulations (EC) No 847/96, (EC) No 2371/2002, (EC) No 811/2004, (EC) No 768/2005, (EC) No 2115/2005, (EC) No 2166/2005, (EC) No 388/2006, (EC) No 509/2007, (EC) No 676/2007, (EC) No 1098/2007, (EC) No 1300/2008, (EC) No 1342/2008 and repealing Regulations (EEC) No 2847/93, (EC) No 1627/94 and (EC) No 1966/2006. Off. J. EU.

[88] (2017). Regulation (EU) No 625/2017 of the European Parliament and of the Council of 15 March 2017 on official controls and other official activities performed to ensure the application of food and feed law, rules on animal health and welfare, plant health and plant protection products, amending Regulations (EC) No 999/2001, (EC) No 396/2005, (EC) No 1069/2009, (EC) No 1107/2009, (EU) No 1151/2012, (EU) No 652/2014, (EU) 2016/429 and (EU) 2016/2031 of the European Parliament and of the Council, Council Regulations (EC) No 1/2005 and (EC) No 1099/2009 and Council Directives 98/58/EC, 1999/74/EC, 2007/43/EC, 2008/119/EC and 2008/120/EC, and repealing Regulations (EC) No 854/2004 and (EC) No 882/2004 of the European Parliament and of the Council, Council Directives 89/608/EEC, 89/662/EEC, 90/425/EEC, 91/496/EEC, 96/23/EC, 96/93/EC and 97/78/EC and Council Decision 92/438/EEC (Official Controls Regulation)Text with EEA relevance. Off. J. EU.

[89] European Parliament (2013). Report on the Food Crisis, Fraud in the Food Chain and the Control Thereof. http://www.europarl.europa.eu/sides/getDoc.do?pubRef=//EP//TEXT+REPORT+A7-2013-0434+0+DOC+XML+V0//EN.www.iucnredlist.org.

[90] Gao Z., Liu Y., Wang X., Wei X., Han J. (2019). DNA mini-barcoding: A derived barcoding method for herbal molecular identification. Front. Plant. Sci..

[91] Shokralla S., Hellberg R.S., Handy S.M., King I., Hajibabaei M. (2015). A DNA mini-barcoding system for authentication of processed fish products. Sci. Rep..

[92] Staffen C.F., Staffen M.D., Becker M.L., Löfgren S.E., Costa Netto Muniz Y., Hajenius Aché de Freitas R., Marrero A.R. (2017). DNA barcoding reveals the mislabeling of fish in a popular tourist destination in Brazil. PeerJ.

[93] Cerutti-Pereyra F., Meekan M.G., Wei N.-W.V., O’Shea O., Bradshaw C.J.A., Austin C.M. (2012). Identification of rays through DNA barcoding: An application for ecologists. PLoS ONE.

[94] Liu S.-Y.V., Chan C.-L.C., Lin O., Hu C.-S., Chen C.A. (2013). DNA barcoding of shark meats identify species composition and CITES-listed species from the markets in Taiwan. PLoS ONE.

[95] Sato Y., Miya M., Fukunaga T., Sado T., Iwasaki W. (2018). MitoFish and MiFish pipeline: A Mitochondrial genome database of fish with an analysis pipeline for environmental DNA metabarcoding. Mol. Biol. Evol..

